# Effect of schistosomiasis on the outcome of patients infected with HIV-1 starting antiretroviral therapy in rural Tanzania

**DOI:** 10.1371/journal.pntd.0006844

**Published:** 2018-10-17

**Authors:** Katarina Stete, Tracy R. Glass, Govert J. van Dam, Alex Ntamatungiro, Emilio Letang, Claudia J. de Dood, Paul L. A. M. Corstjens, Robert Ndege, Herry Mapesi, Winfried V. Kern, Christoph Hatz, Maja Weisser, Jürg Utzinger, Matthias C. Müller

**Affiliations:** 1 Division of Infectious Diseases, Department of Medicine II, University Medical Center Freiburg, Faculty of Medicine, University of Freiburg, Freiburg, Germany; 2 Swiss Tropical and Public Health Institute, Basel, Switzerland; 3 University of Basel, Basel, Switzerland; 4 Department of Parasitology, Leiden University Medical Centre, Leiden, The Netherlands; 5 Ifakara Health Institute, Ifakara Branch, Ifakara, United Republic of Tanzania; 6 ISGlobal, Barcelona Centre for International Health Research, Hospital Clínic, University of Barcelona, Barcelona, Spain; 7 Department of Cell and Chemical Biology, Leiden University Medical Center, Leiden, The Netherlands; 8 St. Francis Referral Hospital, Ifakara, United Republic of Tanzania; 9 Cantonal Hospital St. Gallen, Department of Infectious Diseases and Hospital Epidemiology, St. Gallen, Switzerland; University of Edinburgh, UNITED KINGDOM

## Abstract

**Background:**

It has been hypothesized that schistosomiasis negatively influences immune reconstitution in people living with HIV starting antiretroviral therapy (ART). In this study, we investigated the effect of schistosomiasis on the course of HIV infection in patients starting ART in a rural part of Tanzania.

**Methodology:**

Retrospective study including patients prospectively enrolled in a HIV cohort in Ifakara, south-central Tanzania between January 1, 2013 and April 1, 2015. Schistosomal circulating anodic antigen (CAA) was assessed in pre-ART cryopreserved plasma. Regression models were utilized to estimate the effect of CAA positivity on virological and immunological failure and a composite outcome of death/loss to follow-up (LFU).

**Principal findings:**

At ART-initiation 19.1% (88/461) of patients were CAA-positive. A tendency of higher CD4 increases was seen in CAA-positive patients (+182 cells/μl, interquartile range (IQR), 87–285 cells/μl) compared to CAA-negative patients (+147 cells/μl, IQR, 55–234 cells/μl, p = 0.09) after 10 months of follow-up. After adjustment for baseline risk factors, CAA-positivity showed no association with virological or immunological failure. In CAA-positive patients, 22.7% (20/88) died or were LFU, compared to 29.5% (110/373) of CAA-negative patients (hazard ratio (HR): 0.76, 95% confidence interval (CI), 0.47–1.22, p = 0.25). After adjustment for age, sex, body mass index, educational attainment, WHO-stage, tuberculosis status, and year of ART initiation, CAA-positivity showed a trend of a decreased hazard of death/LFU (HR: 0.58, 95% CI: 0.32–1.05, p = 0.07), while CD4 count at baseline (HR: 0.86, 95% CI: 0.76–1.00, p = 0.02) and MXD (sum of eosinophils, basophils, and monocytes counts) >1,100 cells/μl (HR: 0.56, 95% CI: 0.34–0.93, p = 0.03) were identified as independently protective factors.

**Conclusions/Significance:**

Schistosomiasis is prevalent in this HIV cohort and may be beneficial for immunological reconstitution, while no effect on virological failure was apparent. A positive effect of schistosomiasis-induced immunomodulation on survival and retention in care needs confirmation in future studies.

## Author summary

Infections with HIV and blood flukes (*Schistosoma*) both exert chronic modulatory effects on the host’s immune system. Coinfections, meaning the host is simultaneously infected with both pathogens, are common in sub-Saharan Africa. In this situation the induced immune modulation of one pathogen may affect the course of the disease induced by the other pathogen. One study showed that coinfection with *Schistosoma* in people living with HIV who begin antiretroviral therapy (ART) may have deleterious effects on the reconstitution of the HIV-induced immunosuppression. In the current study, we investigated the effect of *Schistosoma* coinfection on the recovery of the patient’s immune system, on the efficacy of ART to suppress HIV replication, and on a combined endpoint of lost to follow-up or death. We found that schistosomiasis may have beneficial effects on immune reconstitution, while no deleterious effect was detected on HIV-suppressive efficacy of ART. Surprisingly, our data suggest that schistosomiasis-induced immunomodulatory effects might be beneficial for survival and retention in care. Future studies are warranted to confirm these findings. In the era of increasing access to ART in sub-Saharan Africa, the issue of schistosomiasis-HIV coinfection may have major consequences on the outcome of HIV treatment programs.

## Introduction

The geographic distributions of HIV/AIDS and schistosomiasis largely overlap in sub-Saharan Africa, where *Schistosoma* prevalence reaches up to 30% in HIV cohorts [1–4]. In settings of coinfection, *Schistosoma* and HIV mutually interfere on several levels, which may impact the course of the associated diseases [5,6]. As both infections induce chronic modulations of the host’s immune system, interactions on this level are of particular interest [7]. Schistosomiasis and other helminth infections lead to an upregulation of T-helper cell type 2 (Th-2) immune response and a downregulation of T-helper cell type 1 (Th-1) immune response and of cytolytic activity of CD8 T-cells [8,9]. Properties of Th-1 immune response, which includes the secretion of interferon-γ and interleukin (IL)-2 by Th-1 lymphocytes promoting the activation of macrophages and dendritic cells and thereby enhances the ability to kill intracellular pathogens, are essential for the control of viral infections [10]. In line with these immunological findings, a study in an *Ascaris lumbricoides-*HIV coinfected population found higher levels of immune activation, HIV-RNA concentrations, and lower CD4 T-cell counts in individuals with Th-2 bias, as indicated by high *A*. *lumbricoides* fecal egg counts, eosinophilia, and IgE response, compared to patients with high *A*. *lumbricoides* fecal egg counts, low eosinophil count, and low IgE responses [11]. In a study carried out in Zimbabwe, a reduction in viral load with increase of CD4 T-cell count was seen in *Schistosoma*-HIV coinfected patients after antischistosomal treatment [12], and similar effects have been shown after treatment of other helminth infections [13,14].

To our knowledge, only two studies have investigated the effect of *Schistosoma* coinfection on the course of HIV infection in people living with HIV (PLWH) under antiretroviral treatment (ART) [15,16]. Efraim et al. showed that in *Schistosoma*-HIV coinfected patients starting ART, the odds for immunologic treatment failure were four times higher and CD4 cell count increases were significantly lower compared to PLWH without concurrent schistosomiasis [16]. The effect of schistosomiasis on the virological response or on clinical outcomes was not assessed in that study. In settings of high *Schistosoma* prevalence and massive roll out of ART in HIV cohorts, *Schistosoma*-induced treatment failure would have major implications for ART programs.

The working hypothesis of the current study was that *Schistosoma* coinfection has a negative impact on the course of HIV infection in patients starting ART. To test the hypothesis, we aimed to assess the effect of *Schistosoma* coinfection on patients’ response to ART in terms of (i) loss to follow-up (LFU) or mortality; (ii) immunological failure; and (iii) virological failure. The study was conducted in a well characterized HIV cohort in a rural part of south-central Tanzania and employed a highly sensitive diagnostic approach for schistosomiasis.

## Methods

### Ethics statement

The Kilombero and Ulanga Antiretroviral Cohort (KIULARCO) received ethics approval from the institutional review board of the Ifakara Health Institute (IHI) and from the National Health Research Ethics Review Committee of the National Institute for Medical Research of Tanzania. All patients provided written informed consent at inclusion in the KIULARCO cohort and parents or guardians provided informed consent on behalf of all participants under the age of 18 years. We re-tested all circulating anodic antigen (CAA)-positive patients for *Schistosoma* infection. Patients with positive test results were treated with praziquantel (40 mg/kg twice at 4-week interval).

### Study site

This study was carried out in Ifakara, a primarily rural region of south-central Tanzania. Patients were recruited from KIULARCO, an ongoing, open, prospective observational HIV cohort of PLWH followed at the Chronic Disease Clinic in Ifakara (CDCI). Further details of the KIULARCO cohort are given elsewhere [17,18]. Within the cohort, venous blood samples are drawn at routine clinic visits before, 3 months after ART initiation, and every 6 months thereafter. Plasma and cell pellets are cryopreserved at ˗80ºC and ˗20°C on site.

### Study design and participants

The study was a retrospective analysis of cryopreserved plasma samples and of data collected within the existing HIV cohort. The study included all patients who were enrolled in KIULARCO, started ART between January 1, 2013 and April 1, 2015, were older than 15 years, not pregnant, had a CD4 count performed at a maximum of 90 days before to 1 week after ART-start, and had at least one pre-ART plasma sample stored (at a maximum of 30 days before ART-start).

### Diagnostic procedures

The *Schistosoma* infection status of each study participant at the moment of ART initiation was retrospectively assessed by testing 50 μl of pre-ART cryopreserved plasma for the presence of CAA with a lateral flow (LF) test with SCAA20 dry format [19,20]. Negative controls and CAA standard series indicated an assay threshold of 10 pg CAA per ml below which samples were designated as negative. If available, a second sample from 6–13 months after ART-initiation was tested for the presence of CAA. Of note, detection of active schistosomiasis has been simplified by recently developed assays for the detection of schistosomal CAA [19,20]. CAA originates from the gut of adult worms of *Schistosoma mansoni* and *S*. *haematobium* (the two schistosome species endemic in Tanzania) and is shed into the host circulation during active infection. After successful treatment with praziquantel, antigen levels decrease within days [21]. In addition, antigen levels are good indicators for worm burden. CAA is highly stable, and hence, can be detected in cryopreserved plasma samples for years after sample preparation [21–23]. Previously used enzyme-linked immunosorbent assays (ELISAs) as well as the currently used LF tests for detection of CAA in plasma are sensitive (80–95%) and highly specific (98–100%) for the diagnosis of active schistosomiasis, and the recently developed dry format of the latter facilitates usage in laboratories in endemic countries [20,24].

Because isolated eosinophil count in peripheral blood is not part of routine diagnostic procedures in the KIULARCO, we used MXD (mixed cell count: sum of the absolute number of eosinophils, basophils, and monocytes counts) as approximate value. Cut-off for elevated MXD value was set at 1,100 cells/μl [25].

The association between CAA-positivity and virological failure was assessed in a subgroup of study participants. Patients who were continuously CAA-positive (CAA positive before ART-initiation, and CAA positive 6–13 months after ART-initiation) were frequency-matched with two controls (CAA-negative pre-ART and 6–13 months after ART-initiation) for age, CD4 cell count, and tuberculosis status at baseline. In these patients, plasma HIV RNA levels were tested in a cryopreserved plasma sample drawn 6–13 months after ART-initiation.

Plasma HIV RNA from 400 μl plasma was extracted using the NucleoSpin Virus kit (Macherey-Nagel; Oensingen, Switzerland) according to the manufacturer’s protocol. Viral RNA quantification was performed with the Brilliant III Ultra-Fast QRT-PCR Master Mix (Agilent Technologies; La Jolla, CA, United States of America) using the StepOne^TM^ Real-Time PCR System (Applied Biosystems; Foster City, CA, United States of America), with a detection limit of 60 viral RNA copies/ml of plasma. CD4 counts are routinely obtained after staining fresh whole blood samples with labeled antibodies: CD4, CD3, CD8, and CD45 in TruCount tubes (BD FACSCalibur; Franklin Lakes, NJ, United States of America).

### Statistical analysis

Data were extracted from a readily available electronic database of KIULARCO. The baseline was defined as the date of ART initiation. Continuous variables were summarized with medians and interquartile ranges (IQR) and categorical variables with frequencies and percentages.

Association of CAA-positivity at ART-initiation with death or LFU was assessed with a multivariate Cox regression model. LFU was defined as no visit to the outpatient clinic for more than 6 months. The time of the event was defined as the day of the last follow-up visit documented in the database. Results were presented with hazard ratios (HR) and 95% confidence intervals (CIs). The assumption of proportional hazards was confirmed by Schoenfeld’s global test (p = 0.08).

The association of CAA-positivity at ART initiation with immunological failure was assessed with a multivariate logistic regression model. Immunological failure was defined as CD4 count falling below baseline or persistently <100 cells/μl at the time of the first measurement of CD4 cell count ≥6 months after ART-start. Results were presented with odds ratios (ORs) and 95% CIs.

For both models the following variables were considered *a prio*ri as potential co-factors/confounders and were included in the multivariable model (no variable selection was done): age (divided by 10), body mass index (BMI), and CD4 count at baseline (divided by 25) were used as continuous variables. Categorical variable included sex, educational attainment (none and primary school vs. secondary school and college/university), and WHO clinical stages of HIV disease (stage 1 and 2 vs. stage 3 and 4). The variable “active tuberculosis” was defined as diagnosis of tuberculosis at baseline or during follow-up. The variable “MXD value” was dichotomized in normal vs. elevated (>1,100 cells/μl, see above). The variable “year of starting ART” was added to the model used to assess the association of CAA-positivity with death/LFU. The variable “delay to CD4 testing” (i.e., time from ART initiation to measurement of CD4 cell count) was added to the model used to assess the association of CAA-positivity with immunological failure. Linearity of the relationship between continuous explanatory variables and the log odds of the dependent variable was tested by adding polynomial terms of each continuous explanatory variable. Polynomial terms showing a significant association with the log odds of the dependent variable were included in the model.

For the analysis of CAA-positivity as a risk factor for virological failure (defined as HIV RNA concentrations above 1,000 copies/ml after 6–12 months of ART initiation), a logistic regression model was employed, controlling for the following potential co-factors/confounders (defined as above): sex, BMI, educational attainment, WHO clinical stages of HIV disease, MXD value, and delay to HIV RNA testing. Results were presented with ORs and 95% CIs.

Data were anonymized and analyzed using STATA version 12.1 (StataCorp; College Station, TX, United States of America).

## Results

A total of 461 eligible ART-naive PLWH were included in the study. Table 1 summarizes the pre-ART baseline characteristics, stratified by CAA status. Overall, 66.6% of participants were females with a median age at enrolment of 38.2 years (IQR, 32.5–45.8 years). Median BMI was 20.2 kg/m^2^ (IQR, 18.4–22.7 kg/m^2^), and median CD4 count was 173 cells/μl (IQR, 61–299 cells/μl). CAA-positive patients had higher CD4 counts, higher MXD values, and there was a lower proportion of females (Table 1).

**Table 1:** Baseline characteristics of the study population in KIULARCO, Tanzania between January 2013 and March 2015.

Variable	Total	CAA^-^	CAA^+^
N	**461**	**373**	**88**
Age, years—median (IQR)	38.2 (32.5–45.8)	38.7 (32.8–46.4)	37.3 (32.1–44.4)
Female	307 (66.6%)	262 (70.2%)	45 (51.1%)
BMI, kg/m2—median (IQR)	20.2 (18.4–22.7)	20.3 (18.5–22.9)	20.0 (18.1–21.5)
Educational attainment			
None	39 (8.5%)	31 (8.3)	8 (9.1%)
Primary school	399 (86.6%)	322 (86.3%)	77 (87.5%)
Secondary school	21 (4.6%)	18 (4.8%)	3 (3.4%)
College/university	2 (0.4%)	2 (0.5%)	0
CD4 cells/μl–median (IQR)	173 (61–299)	162 (60–294)	195 (63–304)
WHO-stage 3 or 4	235 (51.7%)	192 (51.9%)	43 (50.6%)
Active tuberculosis	89 (19.4%)	73 (19.6%)	16 (18.2%)
MXD cells*			
Cells/μl—median (IQR)	759.5 (527.2–1,133.3)	728.0 (511.0–1,086.4)	973.4 (692.9–1,184.4)
>1,100 cells/μl	112 (26.2%)	83 (24.1%)	29 (34.9%)

*MXD: sum of the absolute number of eosinophils, basophils, and monocytes counts

Data are presented as n (%) or median (IQR)

### Prevalence of schistosomal antigenemia

Among the 461 patients recruited, 88 (19.1%, 95% CI, 15.6–23.0%) were CAA-positive. The median CAA titre at ART initiation was 163 pg/ml (IQR, 27–760 pg/ml). After a median time of 36 weeks on ART (IQR, 29–42 weeks), 36 remained CAA-positive, 17 became CAA-negative, and 35 had no follow-up plasma sample. In the group of 373 initially CAA-negative patients, 214 patients were continuously CAA-negative at ART-initiation and in the follow-up testing after a median time of 35 weeks (IQR, 29–43 weeks), four became CAA-positive, and 155 had no follow-up plasma sample.

### Outcome and predictors of death and LFU

Of 88 CAA-positive patients at ART initiation, 20 (22.7%) died or were LFU, compared to 110 out of 373 (29.5%) of CAA-negative patients (HR: 0.76, 95% CI, 0.47–1.22, p = 0.25). The median survival time in CAA-positive patients was 20.1 months (IQR, 11.9–29.1 months), compared with 19.8 months (IQR, 12.3–26.8 months; p = 0.57) for CAA-negative patients.

**Fig 1:**
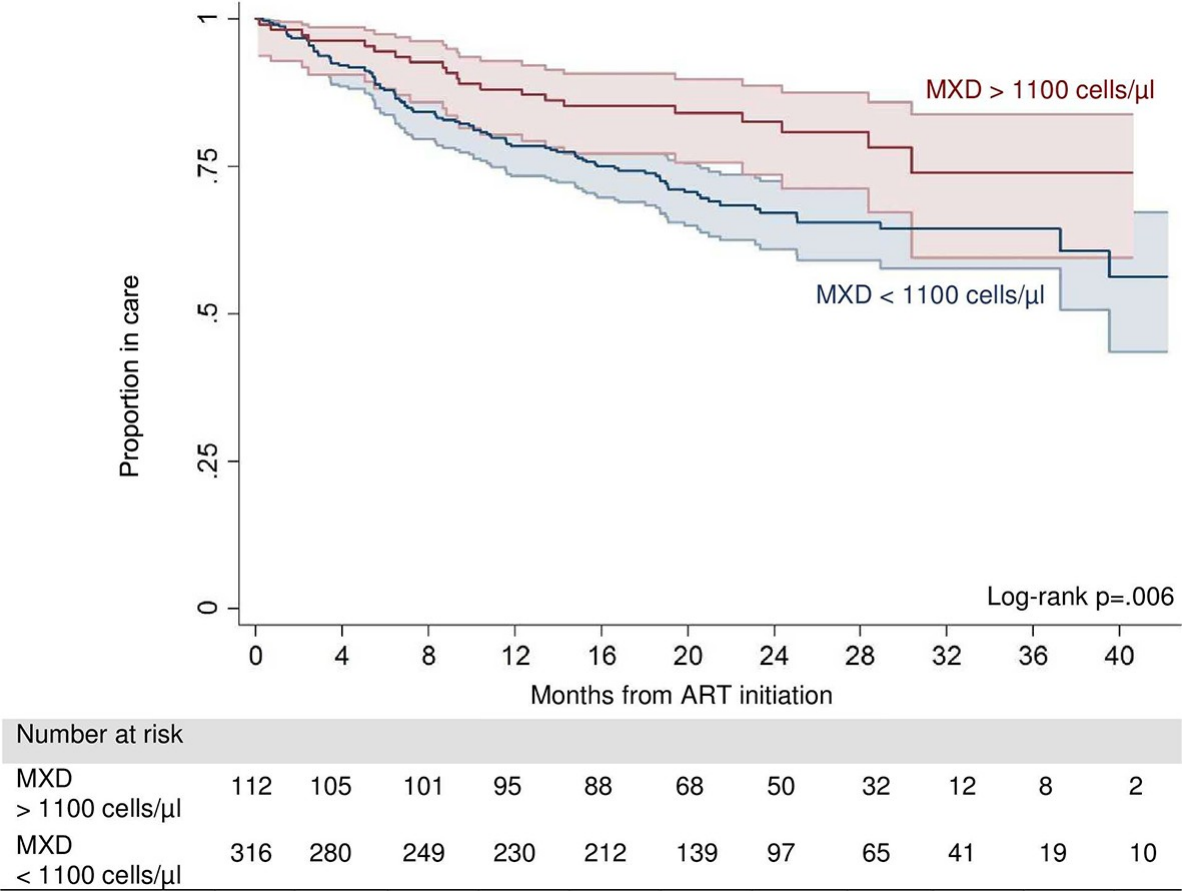
Kaplan-Meier survival estimates of death or loss to follow-up by MXD value (MXD value: Sum of the absolute number of eosinophils, basophils, and monocytes counts).

Baseline risk factors for death or LFU are presented in Table 2. After adjusting for age, sex, BMI, educational attainment, baseline CD4 count, WHO-stage, active tuberculosis, and year of ART initiation, CAA positivity showed a trend of a decreased hazard of death or LFU (HR: 0.58, 95% CI: 0.32–1.05, p = 0.07), while CD4 count and MXD >1,100 cells/μl (HR: 0.56, 95% CI: 0.34–0.93–0, p = 0.03) were identified as independent protective factors (Fig 1).

**Table 2:** Association with death or loss to follow-up, using a Cox regression model.

Variable	Unadjusted/ univariate model		Adjusted/ multivariate model (n = 108 events*)	
	HR (95% CI)	P-value	HR (95% CI)	P-value
CAA-positive	0.76 (0.47–1.22)	0.25	0.58 (0.32–1.05)	0.07
Age, per 10 years	0.96 (0.81–1.12)	0.60	0.98 (0.80–1.19)	0.83
Female	0.75 (0.53–1.07)	0.11	0.76 (0.50–1.15)	0.19
Body mass index (BMI) per kg/m^2^	0.90 (0.85–0.95)	<0.01	0.94 (0.88–1.00)	0.06
Education lower vs. higher**	0.64 (0.26–1.57)	0.33	0.70 (0.28–1.75)	0.45
CD4 cell count, per 25/μl	0.84 (0.75–0.94)	<0.01	0.86 (0.76–1.00)	0.02
CD4 cell count^2^, per 25/μl	1.01 (1.00–1.02)	0.01	1.00 (1.00–1.02)	0.03
CD4 cell count^4^, per 25/μl	1.00 (1.00–1.00)	0.06	1.00 (1.00–1.00)	0.07
WHO-stage 3/4 vs. 1/2	2.37 (1.62–3.45)	<0.01	1.44 (0.90–2.31)	0.13
Active tuberculosis	1.62 (1.08–2.43)	0.02	1.20 (0.74–1.93)	0.46
MXD >1,100 cells/μl***	0.54 (0.34–0.86)	0.01	0.56 (0.34–0.93)	0.03
ART started 2014	1.34 (0.89–2.02)	0.16	1.24 (0.74–2.06)	0.40
ART started 2015	1.91 (1.13–3.22)	0.02	1.75 (0.95–3.22)	0.07

*Event: composite outcome of death/loss to follow-up

**Educational attainment: lower (none/primary) vs. higher than primary

***MXD: sum of the absolute number of eosinophils, basophils, and monocytes counts

### CD4 count reconstitution and immunological failure

A follow-up CD4 count was available for 69 out of 88 (78.4%) of CAA-positive and for 287 out of 373 (76.9%) of CAA-negative patients. CAA-positive patients showed a median difference from baseline in CD4 of +182 cells/μl (IQR, 87–285 cells/μl), compared to +147 cells/μl (IQR, 55–234 cells/μl, p = 0.09) in CAA-negative patients. Median time from ART initiation to measurement of CD4 count was 37.9 weeks (IQR, 32.1–43.7 weeks) for CAA-positive patients, compared to 37.9 weeks (IQR, 28.0–45.1 weeks) in CAA-negative patients. MXD values had no effect on CD4 count changes (MXD <1,100/μl: +151 cells/μl, IQR, 67–236 cells/μl; MXD >1,100/μl: +138 cells/μl, IQR, 51–221 cells/μl, p = 0.60).

Sixty-seven patients (18.8%) met at least one WHO criterion for immunological failure. CAA positivity showed no effect on the risk of immunological failure (OR: 0.78, 95% CI: 0.39–1.59, p = 0.50). Baseline risk factors for immunological failure are presented in Table 3. After adjusting for age, sex, BMI, educational attainment, WHO-stage, active tuberculosis, MXD, and median time from ART initiation to measurement of CD4 count, CAA positivity showed no association with immunological failure (OR: 0.71, 95% CI: 0.32–1.59, p = 0.41), whereas increasing CD4 count at ART initiation was an independent risk factor for immunological failure (OR: 1.08, 95% CI: 1.03–1.13, p = 0.02).

**Table 3:** Association with immunological failure, using a logistic regression model.

Variable	Unadjusted/ univariate model		Adjusted/ multivariate model		
	OR (95% CI)	P-value	OR (95% CI)	P-value	
CAA+	0.78 (0.39–1.59)	0.50	0.71 (0.32–1.59)		0.41
Age, per 10 years	1.09 (0.85–1.39)	0.51	1.04 (0.79–1.37)		0.79
Female	1.18 (0.66–2.12)	0.58	0.75 (0.38–1.50)		0.42
Body mass index (BMI), per kg/m^2^	1.00 (0.93–1.08)	0.97	0.95 (0.86–1.04)		0.28
Education lower vs. higher*	0.22 (0.28–1.64)	0.14	0.28 (0.04–2.21)		0.23
CD4 cell count, per 25/μl	1.08 (1.04–1.13)	<0.01	1.08 (1.03–1.13)		0.02
Delay to CD4 testing**	0.97 (0.95–1.00)	0.07	0.98 (0.95–1.01)		0.26
WHO-stage 3/4 vs. 1/2	0.87 (0.50–1.48)	0.60	0.88 (0.45–1.71)		0.71
Active tuberculosis	0.63 (0.28–1.40)	0.25	0.38 (0.14–1.05)		0.06
MXD >1,100 cells/μl***	1.13 (0.62–2.07)	0.68	1.10 (0.58–2.12)		0.76
Constant			3.35 (0.11–100.72)		0.49

*Educational attainment: lower (none/primary) vs. higher than primary

**Delay to CD4 testing: median time from ART initiation to measurement of CD4 cell count in weeks

***MXD: cumulative value of eosinophils, basophils, and monocytes

### Virological failure

One continuously CAA-positive patient and three continuously CAA-negative patients were excluded from the analysis due to non-availability of plasma samples for HIV PCR. After a median time of 43.4 weeks (IQR, 38.0–49.9 weeks) of ART, eight out of 35 (22.9%) persistent CAA-positive patients had HIV RNA concentrations above 1,000 cp/ml, compared to 12 out of 67 (17.9%) of continuously CAA-negative patients after frequency matching for tuberculosis-status, CD4 count, and age. After adjustment for baseline risk factors, positive CAA status showed no association with virological failure (OR: 1.63, 95% CI: 0.53–4.95, p = 0.39, Table 4).

**Table 4:** Association with virological failure, using a logistic regression model.

Variable	Unadjusted/ univariate model		Adjusted/ multivariate model	
	OR (95% CI)	P-value	OR (95% CI)	P-value
CAA+	0.78 (0.39–1.59)	0.50	1.63 (0.53–4.95)	0.39
Female	0.96 (0.35–2.69)	0.94	0.94 (0.30–2.94)	0.91
Body mass index, per kg/m^2^	1.00 (0.85–1.18)	1.00	1.05 (0.88–1.26)	0.59
Education higher vs. other*	0.67 (0.08–5.87)	0.71	0.78 (0.08–7.57)	0.83
Delay to HIV RNA testing**	1.23 (0.42–3.66)	0.69	0.98 (0.92–1.03)	0.44
WHO-stage 3/4 vs. 1/2	1.45 (0.54–3.89)	0.46	2.34 (0.71–7.77)	0.16
MXD >1,100 cells/μl***	0.47 (0.14–1.54)	0.21	0.47 (0.13–1.63)	0.23
Constant			0.24 (0.00–41.88)	0.58

*Educational attainment: lower (none/primary) vs. higher than primary

**Delay to HIV-RNA testing: median time from ART initiation to measurement of HIV RNA

***MXD: cum sum of the absolute number of eosinophils, basophils, and monocytes counts

## Discussion

To our knowledge this is the first study analyzing the effect of *Schistosoma* coinfection on the immunovirological response and the long-term clinical outcome of PLWH starting ART. Highly prevalent active *Schistosoma* coinfection showed no association with virological and immunological treatment failure but a trend of decreased hazard of death or LFU in patients initiating ART in this mainly rural part of south-central Tanzania. Interestingly, an increase of the MXD value was identified as an independent protective factor against death and LFU in this cohort.

The high prevalence of concomitant active schistosomiasis in this study, as determined by a highly sensitive assay (i.e., CAA) and of schistosomal and helminth infection in general in other studies in PLWH corroborate the potential importance of helminth-HIV coinfection [11,16]. Our findings also highlight that schistosomiasis in adult patients is of special concern as large-scale treatment programs are often school-based and may exclude adults from access to appropriate therapy. An earlier study in KIULARCO yielded a prevalence of 43% for helminth and 11% of *Schistosoma* coinfection determined by stool and urine microscopy (Cornelia Staehelin, unpublished data)[26]. We do not believe that the higher prevalence of schistosomal infection in our study reflects a true increase of prevalence, but a considerably higher sensitivity of the technique of CAA detection in plasma compared to traditional stool microscopy-based diagnostics [24,26,27]. The low sensitivity of widely used microscopic diagnostics in schistosomal infection may be of special concern in settings were HIV co-exists. Schistosomal maturation and egg excretion are thought to depend of the host’s immune response and some studies suggest that HIV-induced CD4 cell depletion may be linked to a decreased luminal migration of schistosome eggs and an arrest of worm development [28–30]. From a clinical point of view, the implication of a possibly reduced sensitivity of microscopy-based diagnostic in HIV-infected individuals would be preclusion from anthelmintic treatment; in clinical trials on *Schistosoma*-HIV coinfection falsely negative tested *Schistosoma* coinfected patients would lead to misclassification and consequently to an underestimation of the effect under investigation. In such circumstances, the use of highly sensitive tests should be considered.

In contrast to the findings of Efraim and colleagues, our study did not show an association of *Schistosoma* coinfection with an elevated risk for immunological failure or decelerated CD4 count gain in PLWH starting ART [16]. Considering evidence from previous research and pathophysiological mechanisms, a detrimental effect of helminth coinfection on CD4 counts recovery may be expected. Our findings, however, are in line with the findings of Muok and colleagues, who also showed a higher increase of CD4 count one month after initiating ART in patients who were infected with *S*. *mansoni*, compared to controls without *Schistosoma* infection [15]. Our results also suggest that elevated pre-ART CD4 cell counts are a risk factor for immunological failure. This counterintuitive association is a well-known phenomenon in European and sub-Saharan cohort studies and is generally explained by a closer clinical monitoring and better adherence of patients with lower CD4 cell count who are at an increased risk for opportunistic infections [31–33].

The rate of virological failure was substantial, which is a frequent problem, especially in rural settings of sub-Saharan Africa [34–36]. Essential factors for a virological successful ART are an adequate regimen and good adherence to therapy. High HIV RNA concentrations prior to initiation of ART are an additional risk factor for virological treatment failure [37]. Because helminth-induced immunomodulatory effects are associated with elevated HIV RNA levels in ART-naïve individuals, helminth infection could theoretically be associated with an elevated risk of virological failure [11]. Our study did not show an impact of schistosomal infection on virological treatment failure, probably because potential differences in HIV RNA concentrations prior to ART initiation are not important enough to affect efficacy of ART.

The protective effect of schistosomiasis in PLWH starting ART in our study is counterintuitive. Possible explanations of the results include immunomodulatory properties of *Schistosoma* spp., which may exert beneficial effects in the setting of ART-induced immunological reconstitution. Immune reconstitution inflammatory syndrome (IRIS) is a condition seen in PLWH with profound CD4 T cell depletion and is characterized by an overwhelming inflammatory response to pathogens due to a recovering T cell count after initiation of ART. IRIS is a common cause of early mortality of patients starting ART in sub-Saharan Africa [38]. *Schistosoma*-induced immunomodulation may have an attenuating effect on IRIS-related morbidity and mortality and by this means, improve the clinical outcome of PLWH starting ART.

The statistically significant association of elevated MXD values with a reduced risk of death and LFU may support this assumption. MXD value is composed of absolute cell counts of monocytes, eosinophilic, and basophilic granulocytes, which are independent from HIV infection and disease status [39–41]. In the current setting in a primarily rural area of south-central Tanzania, elevated MXD values are most likely driven by helminth-induced eosinophilia and may be construed as a surrogate marker of helminth-induced immunomodulation in the HIV-infected host.

Preceding studies in KIULARCO identified an important burden and variety of helminthic infections. Because immunomodulatory effects are a consistent feature of helminth infection, we believe that the weaker effect of schistosomal infection on survival/retention in care in our study may possibly be explained by competing immunomodulatory effects of other undetected helminths as our diagnostics in this study were focused on schistosomiasis. Indeed, about a quarter of PLWH without *Schistosoma* infection had elevated MXD values.

Our study has several shortcomings that are offered for discussion. First, clinical data of KIULARCO are collected prospectively, but CAA was identified retrospectively in cryopreserved plasma samples with the immanent weaknesses of a retrospective study design. Second, due to a suboptimal ascertainment of mortality in our cohort, we employed a composite outcome of death/LFU, limiting our conclusions regarding the impact of schistosomiasis on mortality. However, LFU is a common issue in ART programs in resource-limited settings and mortality is inversely associated with the rate of LFU [42]. On the basis of an overall LFU of approximately 20% in KIULARCO, mortality of patients and LFU is expected to be about 50% [42]. Third, confounding factors cannot be excluded. In particular, schistosomiasis and elevated MXD values could be associated with other characteristics of the study population, which reduce mortality or LFU, remained unrecognized for which we could not control for (e.g., distance to the clinic or migratory mobility). Fourth, because pre-ART HIV-RNA concentrations were not assessed, it cannot be excluded that uneven distributions of HIV-RNA concentrations between both groups may have introduced bias in the presented results for the effect of CAA-positivity on the virological outcome.

In conclusion, testing for CAA in plasma revealed a 19.1% prevalence of active schistosomiasis in PLWH starting ART in the KIULARCO in rural south-central Tanzania. This observation corroborates concerns of limited sensitivity of microscopy-based diagnostics, which might be additionally compromised in HIV-infected populations and may impede treatment for patients with a potentially life-threatening disease [43]. Our study could not detect detrimental properties of *Schistosoma* coinfection on immunological and virological response to ART but suggests that helminth-induced immunomodulatory mechanisms might enhance survival of PLWH starting ART.

Our results need confirmation in future studies, which should consider (i) the dynamic aspect of HIV infection and therefore preferentially chose a longitudinal study design, including clinical outcomes; and (ii) that individuals are often coinfected by multiple parasitic worms that may affect the course of the HIV infection by comparable but interindividually variable immunomodulatory effects. To avoid misclassification, comprehensive, highly sensitive helminth diagnostics and markers of the host’s immunological response should be employed. Among these highly sensitive tests, the used CAA-test is a promising candidate and further use and developments may improve the acceptance as a future standard test.

In the era of massive rollout of ART in sub-Saharan Africa and other tropical and sub-tropical countries, the issue of helminth-HIV coinfection may have major ramifications on the outcome of HIV treatment programs. Further research, especially in PLWH initiating ART, is urgently needed.

## Supporting information

S1 STROBE Checklist (DOC)

S1 Dataset (DTA)
