# Supplementary material for: Effect of schistosomiasis on the outcome of patients infected with HIV-1 starting antiretroviral therapy in rural Tanzania
Source: PLoS Negl Trop Dis. 2018 Oct 17;12(10):e0006844. doi: 10.1371/journal.pntd.0006844 (PMC6205655; doi:10.1371/journal.pntd.0006844)
Supplement: S1 STROBE Checklist — (DOC) [file pntd.0006844.s001.doc]

STROBE Statement—Checklist of items that should be included in reports of ***cohort studies***

|  | Item No | Recommendation |
| --- | --- | --- |
| **Title and abstract** | 1 | (*a*) Indicate the study’s design with a commonly used term in the title or the abstract. Abstract: First sentence |
|  |  | (*b*) Provide in the abstract an informative and balanced summary of what was done and what was found: Abstract: Methodology/Findings |
| Introduction | | |
| Background/rationale | 2 | Explain the scientific background and rationale for the investigation being reported  Introduction: First and second paragraph |
| Objectives | 3 | State specific objectives, including any prespecified hypotheses  Introduction: Third paragraph |
| Methods | | |
| Study design | 4 | Present key elements of study design early in the paper  Page 6: Study design and diagnostic procedures |
| Setting | 5 | Describe the setting, locations, and relevant dates, including periods of recruitment, exposure, follow-up, and data collection  Page 6: Study setting /Study participants  Page 8, Statistical analysis: second sentence. |
| Participants | 6 | (*a*) Give the eligibility criteria, and the sources and methods of selection of participants. Describe methods of follow-up Page 6: Study setting /Study participants |
|  |  | (*b*) For matched studies, give matching criteria and number of exposed and unexposed  Page 7, Study design and diagnostic procedures: Third paragraph |
| Variables | 7 | Clearly define all outcomes, exposures, predictors, potential confounders, and effect modifiers. Give diagnostic criteria, if applicable  Page 8: Statistical analyisis |
| Data sources/ measurement | 8* | For each variable of interest, give sources of data and details of methods of assessment (measurement).  Page 8, Statistical analyisis: First paragraph  Page 6, Study design and diagnostic procedures: First and fourth paragraph |
| Bias | 9 | Describe any efforts to address potential sources of bias  Page 8, statistical analysis: reduce risk of confounding by using a multivariate regression analysis with variables which were considered as potential confounders  Page 6: Study design and diagnostic procedures, first paragraph: avoidance of misclassification by using a highly sensitive and specific diagnostic tool (CAA-LFA). |
| Study size | 10 | Explain how the study size was arrived at  Page 6, Study participants: Second sentence |
| Quantitative variables | 11 | Explain how quantitative variables were handled in the analyses.  Page 7, Study design and diagnostic procedures: second paragraph  Results: Table 2/Table 3/Table 4 |
| Statistical methods | 12 | (*a*) Describe all statistical methods, including those used to control for confounding  Page 8: Statistical analysis |
|  |  | (*b*) Describe any methods used to examine subgroups and interactions  Page 7, Study design and diagnostic procedures: third paragraph |
|  |  | (*c*) Explain how missing data were addressed N/A |
|  |  | (*d*) If applicable, explain how loss to follow-up was addressed  Page 8: Statistical analysis: second paragraph |
|  |  | (*e*) Describe any sensitivity analyses : N/A |
| Results | | |
| Participants | 13* | (a) Report numbers of individuals at each stage of study—eg numbers potentially eligible, examined for eligibility, confirmed eligible, included in the study, completing follow-up, and analysed  Results: first paragraph, paragraphs: ”Prevalence of antigenemia”, ”Outcome and predictors of death and LFU”, “CD4 count reconstitution and immunological failure”, and “Virological failure” |
|  |  | (b) Give reasons for non-participation at each stage  Please refer to 13a) |
|  |  | (c) Consider use of a flow diagram  N/A |
| Descriptive data | 14* | (a) Give characteristics of study participants (eg demographic, clinical, social) and information on exposures and potential confounders  Results: First paragraph and Table 1 |
|  |  | (b) Indicate number of participants with missing data for each variable of interest  N/A |
|  |  | (c) Summarise follow-up time (eg, average and total amount)   - Outcome and predictors of death and LFU: First paragraph - CD count reconstitution and immunological failure: First paragraph - Virological failure: First paragraph |
| Outcome data | 15* | Report numbers of outcome events or summary measures over time   - Outcome and predictors of death and LFU: First paragraph - CD count reconstitution and immunological failure: Second paragraph - Virological failure: First paragraph |
| Main results | 16 | (*a*) Give unadjusted estimates and, if applicable, confounder-adjusted estimates and their precision (eg, 95% confidence interval). Make clear which confounders were adjusted for and why they were included   - Outcome and predictors of death and LFU: First paragraph and Table 2 - CD count reconstitution and immunological failure: First paragraph and Table 3 - Virological failure: First paragraph and Table 4 |
|  |  | (*b*) Report category boundaries when continuous variables were categorized  Refer to Table 1 to Table 4 |
|  |  | (*c*) If relevant, consider translating estimates of relative risk into absolute risk for a meaningful time period  N/A |
| Other analyses | 17 | Report other analyses done—eg analyses of subgroups and interactions, and sensitivity analyses  Refer to Virological failure |
| Discussion | | |
| Key results | 18 | Summarise key results with reference to study objectives  Discussion: First paragraph |
| Limitations | 19 | Discuss limitations of the study, taking into account sources of potential bias or imprecision. Discuss both direction and magnitude of any potential bias  Discussion: Paragraph 8 |
| Interpretation | 20 | Give a cautious overall interpretation of results considering objectives, limitations, multiplicity of analyses, results from similar studies, and other relevant evidence  Discussion: Paragraph 1-8 |
| Generalisability | 21 | Discuss the generalisability (external validity) of the study results  Discussion: Paragraph 9-10 |
| Other information | | |
| Funding | 22 | Give the source of funding and the role of the funders for the present study and, if applicable, for the original study on which the present article is based  Please refer to the section “Additional Information” of the online submitting system |

*Give information separately for exposed and unexposed groups.

**Note:** An Explanation and Elaboration article discusses each checklist item and gives methodological background and published examples of transparent reporting. The STROBE checklist is best used in conjunction with this article (freely available on the Web sites of PLoS Medicine at http://www.plosmedicine.org/, Annals of Internal Medicine at http://www.annals.org/, and Epidemiology at http://www.epidem.com/). Information on the STROBE Initiative is available at http://www.strobe-statement.org.
